# Teaching and learning the mental state exam in an integrated medical school. Part II: Student performance

**DOI:** 10.1192/pb.bp.113.042663

**Published:** 2014-10

**Authors:** Sarah Huline-Dickens, Eithne Heffernan, Paul Bradley, Lee Coombes

**Affiliations:** 1 Peninsula College of Medicine and Dentistry, Plymouth, UK

## Abstract

**Aims and method** To investigate medical students’ performance at and perceptions of the mental state examination (MSE) at a medical school with a modern integrated curriculum. We undertook an evaluative case study comprising a survey and analysis of performance data. The study is presented in two parts: part 2 reports the students’ performance data as assessed by integrated structured clinical examination (ISCE).

**Results** About a third of students (32.7%) thought that the MSE ISCE was more difficult than the non-MSE ISCE from the questionnaire data. The evidence from the ISCE performance data indicates that there are no significant differences between the scores of students in the MSE station and the non-MSE stations.

**Clinical implications** Most studnets do not find the MSE ISCE station more difficult than other ISCE stations. Perhaps therefore students should be reassured that assessments in psychiatry are just like other assessments in medicine. For some students, however, performing at the MSE ISCE station is a more complex challenge.

Little is written about the mental state exam (MSE) and how it is taught in medical schools. This is the second of two papers on the teaching and learning of the MSE among undergraduate medical students. The first paper chiefly concerned students’ perceptions of the MSE: this paper focuses on the performance of students at the integrated structured clinical examination (ISCE).

Methods of teaching and learning in psychiatry vary between medical schools and are difficult to compare, but the objective structured clinical examination (OSCE) is a common approach to assessment. The OSCE, introduced in 1975 by Harden *et al*^1^ and further described in Harden & Gleeson^2^ in 1979 is now very widely used in the assessment of clinical competence employing real patients, simulated patients and other forms of patient substitute.^3^ The principles of its use are now well established. The OSCE is an instrument for the assessment of clinical competence and assesses the ‘shows how’ level of Miller’s pyramid of clinical competence.^4^ The reliability, validity and feasibility of the OSCE are well described in a consensus statement of medical educationalists.^5^ Scores can be generated for high-stakes examination purposes enabling justifiable and defensible decisions and facilitating feedback on performance.

There is supportive evidence for good reliability, validity and feasibility of the OSCE in psychiatry, although some authors harbour doubts about its limitations in the specialty. In a review of the literature, it is noted that OSCEs in psychiatry may not capture the nuances of psychiatric interview, that the time limitations are too severe, and that psychiatric OSCEs may not allow for patient complexity.^6^

## Assessment strategy at Peninsula Medical School

At Peninsula Medical School the OSCE has been adapted to include communication skills, patient-centred issues and professional behaviours that are consistent with *Good Medical Practice*,^9^ in addition to the standard history, examination, investigation, management plan and links to prior learning. It is thus known as an ISCE (integrated structured clinical examination). The ISCE, by incorporating elements of the long case with a real or simulated patient, aims at increasing validity by representing clinical situations more authentically. Students are assessed across a range of variables and are marked using performance criteria corresponding to four grades: unsatisfactory, borderline, satisfactory or excellent.^10^ The assessment strategy at the medical school is described elsewhere.^11^ In year 2, each ISCE station is 25 min. In year 4, each station is 45 min, which includes 20 min of patient interaction, 5 min preparation and 20 min for presenting to the assessor. The ISCE has 12 stations but students who meet the required standard in the first 6 stations are not required to complete the remaining 6 stations.

Common features to both year 2 and year 4 ISCEs are one assessor per station on the basis that two would be too costly and would not add appreciably to reliability. Assessors are usually non-experts and are briefed about the examination in advance of the ISCE for 1 h in a training session. The patients are given scripts and a briefing session independently. The MSE ISCE station tends to use actors, whereas the non-MSE ISCE stations use a combination of actors and real patients.

## Method

The methodology of this study, part of a larger naturalistic study of students’ perceptions of MSE, is described in the companion paper.^12^ Ethical approval was granted for the collection of data by questionnaire but not for linking any performance data with the questionnaire data. It was therefore not possible to assess whether those students who perceived the MSE station to be more difficult actually have a lower performance in this station.

The particular study questions were:

1. Do students perceive their performance in the ISCE stations where they are expected to undertake a mental state exam (MSE ISCE stations) to be lower than their performance in ISCE stations where they are not expected to undertake a mental state exam (non-MSE ISCE stations), based on their responses to a questionnaire?
2. Is the actual performance of students in the MSE ISCE station lower than their performance in the non-MSE ISCE station, based on their percentage scores?
3. If students’ perceived performance is lower in the MSE ISCE stations than in the non-MSE ISCE stations, why might this be?

### Measures

A questionnaire was devised (described in our companion paper and available as an online supplement to that paper)^12^ to answer the first and third research questions about students’ perceptions of their performance in MSE ISCE and non-MSE ISCE stations and the reasons for that. To answer the second research question about the actual performance of students, we gathered ISCE performance data from 2009, 2010 and 2011, comprising a total of 6 iterations of the ISCE exam in years 2 and 4. Only data from the first six stations of each ISCE were used, as few students needed to complete the remaining six stations.

### Data analysis

**Fig 1:**
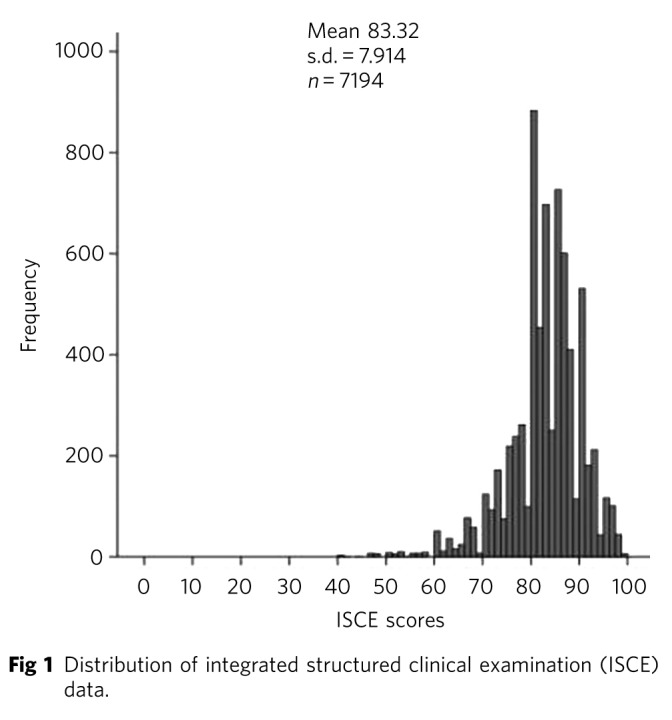
Distribution of integrated structured clinical examination (ISCE) data.

The data were organised using Microsoft Excel 2007 and were analysed using PASW Statistics for Windows, Version 18.0. A Kolmogorov-Smirnov test of normality demonstrated that the ISCE data were not normally distributed (D_(7194)_=0.11, *P*<0.001) (Fig. 1). Therefore, non-parametric tests were used for data analysis. Descriptive statistics were also calculated for both the ISCE and the questionnaire data.

## Results

### Analysis of ISCE performance data

**Fig 2:**
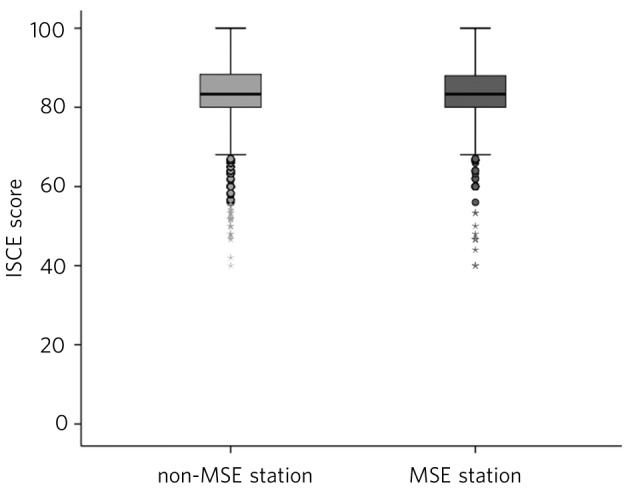
Mental state examination (MSE) and non-MSE stations. ISCE, integrated structured clinical examination.

The descriptive statistics show that there is only a slight difference between scores in the MSE station (mean = 82.83, s.d. = 8.47) and scores in the non-MSE stations (mean = 83.42, s.d. = 7.8) (Table 1 and Fig. 2).

**Fig 3:**
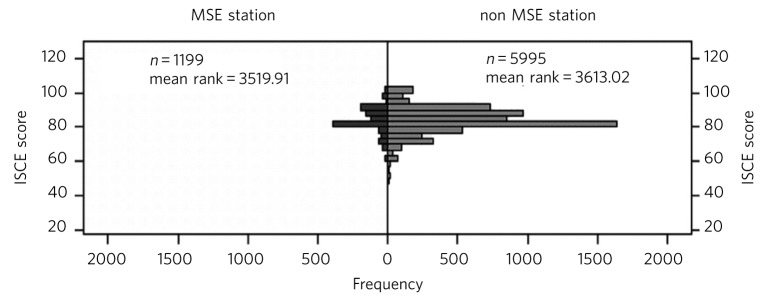
Mann-Whitney *U*-test of mental state examination (MSE) and non-MSE stations. ISCE, integrated structured clinical examination.

An independent samples Mann-Whitney *U*-test was carried out to examine whether students’ performance on MSE stations is significantly different to their performance on non-MSE stations (Fig. 3). The results show that non-MSE station scores were not significantly different to MSE station scores (median 3613.02 *v*. 3519.91 respectively, *U* = 3500970, *P* = 0.156).

### Analysis of questionnaire data

Questionnaires were collected from 229 students out of a possible 342 students in the academic year 2010-11, which gives a response rate of 67%. The characteristics of the students are described in our companion paper.^12^ Results show that the majority of students had positive attitudes towards the MSE ISCE but that a substantial proportion had negative attitudes (Table 2).

Question 27 (‘I think the MSE ISCE station is more difficult than the other ISCE stations’) is crucial to this study as it tests the hypothesis that medical students perceive the MSE ISCE station to be more difficult than the other ISCE stations. The results show that most students (44.1%) disagreed or strongly disagreed with the statement that the MSE station is more difficult. However, nearly a third of students (32.7%) had the opposite view and thought that the MSE ISCE is more difficult. When invited to elaborate on their answers to question 27 in the next question, 68 students gave a total of 99 comments (up to 3 comments each). The most common reasons are listed in Table 3 (note that when actors and timing were mentioned, these are counted twice; this occurred on two occasions).

**Table 1:** Descriptive statistics of percentage scores in the MSE and non-MSE stations

	non-MSE station	MSE station
*N*	5995	1199
		
Mean (95% CI)	83.42 (83.22-83.62)	82.83 (82.35-83.31)
		
Median	83.33	83.33
		
s.d.	7.8	8.47
		
Range	60 (40-100)	60 (40-100)
		
IQR	8.33	8

MSE, mental state examination; IQR, interquartile range.

When the students were asked in question 29 about their peers’ perceived attitudes to MSE ISCE, the majority (65.9%) thought that their peers would find the MSE to be more difficult than the other ISCE stations and 139 students elaborated on that in a follow-up question (182 responses, up to 3 comments each) (Table 4).

## Discussion

The evidence from the ISCE performance data indicates that there are no significant differences between the scores of students in the MSE station and the scores of students in the non-MSE stations. This is supported by evidence from the questionnaire survey, which shows that most students do not perceive the MSE station to be more difficult than the other ISCE stations. The majority of students thought the expectations of what they had to do in the ISCE were clear, the timing was about right and the use of simulated patients was helpful. Views about how realistic the MSE ISCE is were more mixed, with almost equal numbers agreeing, disagreeing or abstaining.

However, although most students believe that the MSE stations are no more challenging than non-MSE stations, the questionnaire data show that about a third of students perceive the MSE stations to be more difficult. Also, the majority of students indicated that their peers would find the MSE station more difficult than the other stations. This is an interesting finding and worthy of some comment.

**Table 2:** Responses to section 3

		%				
Question	*n*	Strongly disagree	Disagree	Don’t know	Agree	Strongly agree
23 The expectations of what students need to do in the MSE ISCE are clear	224	3.1	22.7	15.3	49.8	7
						
24 The time allotted to the MSE ISCE is about right	223	6.6	21	14.8	48	7
						
25 It is helpful to use simulated patients in the MSE ISCE	220	3.9	7	12.2	52.4	20.5
						
26 The MSE ISCE is not realistic	223	2.2	32.8	30.6	25.8	6.1
						
27 The MSE ISCE station is more difficult than the other ISCE stations	224	4.8	39.3	21	25.3	7.4

ISCE, integrated structured clinical examination; MSE, mental state examination.

**Table 3:** Reasons for the increased difficulty of the MSE ISCE (*n* = 99)

Reason	Frequency, %	Illustrative quotes
*MSE*		
The nature of the MSE	9	Many more questions to remember to ask
Teaching, learning and practising the MSE	9	Little teaching in psychiatry means that it is difficult to know what to ask, for example need to know a little bit about OCD to ask good questions
		
*ISCE*		
Timing	22	For the MSE station the history is usually led by the patient and can take longer than the allocated time. This is especially true if the patient is depressed (talks slow, if at all) or is manic and easily distracted
Actors	20	Actors take it too far if they don’t want to answer any of your questions, they won’t no matter what you do
Examiners	3	Although there is enough time, it is dependent on marker
Other	1	Difficult and probably not completely appropriate to do full mental state exam on acute confusional state patient in ISCE
		
*Other*		
Positive comments	6	I find psychiatry interesting, hence I enjoyed learning about it
Not otherwise classified	29	The patient can sometimes be unnerving and you are talking to them for the whole 25 min
		The emphasis is placed entirely on your ability to talk to the patient with no skills required, leaving you more exposed
		It has less of a definitive goal than other sessions
		Because psychiatry and mental health illness is sensitive, personal and challenging
		It is often hard to keep a structure

ISCE, integrated structured clinical examination; MSE, mental state examination; OCD, obsessive-compulsive disorder.

**Table 4:** Reasons for the increased difficulty of the MSE ISCE for peers (*n* = 182)

Reason	Frequency *n* (%)	Illustrative quotes
*MSE*		
The MSE	28 (15)	Some of the words in MSE are entirely new concepts
Teaching, learning and practising the MSE	50 (27)	Insufficient practice, especially for those who struggle with communication
		
*ISCE*		
Timing	21 (11.4)	Not enough time for a good mental health assessment
Actors	23 (12.6)	Actors can be more uncooperative than necessary
Examiners	1 (0.5)	Because each examiner will want different questions asked
Other	14 (7.7)	Not as difficult only because patients and setting is unrealistic, and fewer expectations, lower standard of competence required
		
*Other*		
Positive comments	1 (0.5)	Psychiatry teaching at Peninsula Medical School is adequate
Not otherwise classified	44 (24)	Some students find learning tests in medicine and surgery easier and find sensitive communication more difficult
		Not as black-and-white diagnoses
		Difficult skill to master
		Have to find out about the whole person, not just a physical complaint
		Many people are not into psychiatry and may spend less time on it due to low motivation

ISCE, integrated structured clinical examination; MSE, mental state examination.

One possible paradigm of interpretation may be the so-called ‘better than average’ effect. This is a kind of self-serving bias, and refers to the tendency of people to rate themselves as higher on positive attributes (and lower on negative attributes) than other people. Many studies have considered the fact that people use different information when evaluating themselves compared with evaluating others, and this is discussed further by Williams & Gilovich.^13^ The finding that most students think their peers find the MSE ISCE more difficult than the non-MSE ISCE may also reflect a negative perception of the MSE station, in keeping with the anecdotal belief that the station is harder than the others. The reasons why the MSE is seen, at least by some, as more difficult than other stations were explored.

Most responses (*n* = 29), as indicated in Table 3, fell into a group that were difficult to classify and five examples of these responses are given. These statements illustrate personal discomfort, misgivings about interpretations, a lack of direction, goal and structure, and a sense of challenge in the interview. The next largest category concerned timing and actors. Finally, the next most numerous comments concerned the nature of the MSE and affirmed problems with remembering it. When asked about the experiences of their peers, most students appear to attribute their belief their peers would have difficulty to a lack of practice (*n* = 50, Table 4). The next most numerous category of response, however, were statements that were not easily classified, examples of which are given in the table. Finally, in order of frequency were comments about the MSE itself.

In contrast to the responses to closed-ended questions, the open-ended questions allowed to collect more finely tuned responses. These showed that there were a number of students who raised personal discomfort, problems with timing and actors as a reason for finding the MSE ISCE more difficult as well as a smaller number finding the nature of the mental state exam difficult.

The comments about actors tended to be about students’ perception of overacting or being difficult rather than concerns about ability to empathise with them. This is in keeping with one of the disadvantages of the use of simulated patients listed by Eagles *et al*.^7^ There are many advantages to using simulated patients in assessments of students in psychiatry, including their ready standardisation for consistency purposes, the protection against harmful or repeated use of real patients, and the fact that they can participate in giving feedback to students. Although less has been recorded about the disadvantages, simulated patients may draw on their own experiences, divert from the script, and can overact or be opinionated.^7^ However, in a group of randomly mixed simulated patients and real patients in an assessed interview setting it was found that both students and faculty showed a strong preference for real patients owing to the problem of developing empathy with the simulated patients.^8^

In the quantitative data, only just over half (55%) thought the timing was about right (Table 2), but in responses to open questions the lack of time was a persistent theme mentioned by students. This is in line with the findings by Park *et al*.^6^

### Strengths and limitations

One of the strengths of this study has been the high response rate and the understanding acquired through the collection of quantitative and some qualitative data. The study findings, although limited to one medical school, will be of interest to other medical schools with integrated curricula, as the requirement for newly graduating doctors to be able to perform an MSE is a nationally expected norm.

The study had some limitations. First, the ISCE data were not normally distributed so non-parametric tests, rather than more powerful parametric tests, were used for data analysis. Second, many students selected the ‘don’t know’ option on each question, which results in data that are difficult to interpret. It is sometimes argued that neutral mid-points and ‘don’t know’ options should not be included in questionnaires as respondents may chose this option because it requires little cognitive effort and not because they truly do not know, but equally, ‘don’t know’ options can be valuable because they ensure that respondents are not forced to select ‘agree’ or ‘disagree’ when they truly have no opinion.^14^ A ‘don’t know’ option is especially suited for respondents with a high level of cognitive ability, who are less likely to use them to avoid exerting cognitive effort.

Third, it should be noted that fewer students responded to the open-ended questions than to the closed-ended questions. Finally, the study did not have ethics approval to match the students’ questionnaire data to their ISCE performance data. Therefore, it was not possible to assess whether those students who perceive the MSE station to be more difficult actually have a lower performance in this station. However, there are disadvantages to requiring students to identify themselves in order to link their questionnaire responses to their ISCE scores. For example, some students may change the way in which they respond to the questionnaire or may decide not to complete it, which leads to a smaller and possibly more biased sample.

In spite of these limitations this study has illustrated some interesting points about the perceptions and performance of students at an integrated medical school with a modern curriculum. These can be used locally to inform changes in teaching, learning and assessment practices and perhaps reassure students that assessments in psychiatry are just like other assessments in medicine.

The evidence from the ISCE performance data indicates that there are no significant differences between the scores of students in the MSE station and the scores of students in the non-MSE stations. The ISCE performance data and the questionnaire data together suggest that the anecdotal evidence that the MSE is difficult and challenging for students may be unfounded, but that a number of students might find the task more complex.
